# Children’s Exposure to PBDEs: Binational Comparison Highlights Dramatic Differences

**DOI:** 10.1289/ehp.119-a442a

**Published:** 2011-10-01

**Authors:** Kellyn S. Betts

**Affiliations:** Kellyn S. Betts has written about environmental contaminants, hazards, and technology for solving environmental problems for publications including *EHP* and *Environmental Science & Technology* for more than a dozen years.

Elevated exposures to polybrominated diphenyl ether (PBDE) flame retardants have been linked to developmental neurotoxicity in children and endocrine disruption in adults. A growing body of evidence shows that children’s bodies have higher levels of PBDEs than adults’, possibly because of greater hand-to-mouth activity. One of the largest studies to date of PBDE uptake in children provides important evidence that U.S. children may take in significant amounts of these compounds from their environment [*EHP* 119(10):1442–1448; Eskenazi et al.].

The children were participants in the Center for the Health Assessment of Mothers and Children of Salinas (CHAMACOS) cohort study. Their Mexican-American mothers were recruited to the study through prenatal clinics serving low-income farm workers in California’s Salinas Valley. A team led by researchers at the University of California, Berkeley, School of Public Health assessed measurements of PBDEs in blood serum collected from mothers during pregnancy and from children at age 7 years. On average, the PBDE concentrations in the 264 children were 3 times higher than those measured in their mothers during pregnancy. This suggests that most of the children’s PBDE exposure did not occur prenatally or through breastfeeding.

The researchers then compared the California children’s PBDE levels with those of 283 5-year-olds living in the Mexican states from which the CHAMACOS mothers had come. The Mexican data were collected via a second study, Proyecto Mariposa, which is affiliated with CHAMACOS. The California children’s PBDE levels were, on average, 7 times higher than those of the Mexican children.

The California children’s geometric mean for the seven most commonly found PBDE compounds was 87.8 ng/g serum (lipid weight), levels the researchers say are high enough to “present a major public health challenge” according to evidence of potential health effects of these compounds. In the small body of literature published to date, only children living and working on hazardous waste sites in Nicaragua have been documented to have higher PBDE levels than children in California.

PBDEs have been used extensively in long-lived consumer goods, particularly in California, where the law requires that flame retardants be added to the polyurethane foam used in upholstered furniture and children’s products such as car seats. Many products containing now-banned PBDEs are believed to still be in use throughout the United States and the world.

Because the retardants are not chemically bound to the foam and plastic to which they were added, they can escape into household dust. Although this study did not assess exposure pathways, the results support earlier evidence that inadequate ventilation and deteriorating PBDE-treated foam from aging or poorly maintained furniture may contribute to disproportionately high PBDE exposures in lower-income homes.

